# Clustered randomised controlled trial of two education interventions designed to increase physical activity and well-being of secondary school students: the MOVE Project

**DOI:** 10.1136/bmjopen-2015-009318

**Published:** 2016-01-06

**Authors:** Peter B Tymms, Sarah E Curtis, Ash C Routen, Katie H Thomson, David S Bolden, Susan Bock, Christine E Dunn, Ashley R Cooper, Julian G Elliott, Helen J Moore, Carolyn D Summerbell, Paul A Tiffin, Adetayo S Kasim

**Affiliations:** 1 School of Education, University of Durham, Durham, UK; 2 Department of Geography, University of Durham, Durham, UK; 3 National Centre for Sport & Exercise Medicine (NCSEM), School of Sport, Exercise, and Health Sciences, Loughborough University, Loughborough, UK; 4 School of Applied Social Sciences, University of Durham, Durham, UK; 5 Centre for Exercise, Nutrition and Health Sciences, University of Bristol, Bristol, UK; 6 National Institute for Health Research, Bristol Biomedical Research Unit in Nutrition, Diet and Lifestyle, Bristol, UK; 7 School of Medicine, Pharmacy and Health, University of Durham, Durham, UK; 8 Wolfson Research Institute for Health and Wellbeing, University of Durham, Stockton-on-Tees, UK

**Keywords:** MENTAL HEALTH, SPORTS MEDICINE

## Abstract

**Objective:**

To assess the effectiveness of 2 interventions in improving the physical activity and well-being of secondary school children.

**Design:**

A clustered randomised controlled trial; classes, 1 per school, were assigned to 1 of 3 intervention arms or a control group based on a 2×2 factorial design. The interventions were peer-mentoring and participative learning. Year 7 children (aged 11–12) in the peer-mentoring intervention were paired with year 9 children for 6 weekly mentoring meetings. Year 7 children in the participative learning arm took part in 6 weekly geography lessons using personalised physical activity and Global Positioning System (GPS) data. Year 7 children in the combined intervention received both interventions, with the year 9 children only participating in the mentoring sessions.

**Participants:**

1494 year 7 students from 60 schools in the North of England took part in the trial. Of these, 43 students opted out of taking part in the evaluation measurements, 2 moved teaching group and 58 changed school. Valid accelerometry outcome data were collected for 892 students from 53 schools; and well-being outcome data were available for 927 students from 52 schools.

**Main outcome measures:**

The primary outcomes were mean minutes of accelerometer-measured moderate-to-vigorous intensity physical activity per day, and well-being as evaluated by the KIDSCREEN-27 questionnaire. These data were collected 6 weeks after the intervention; a 12-month follow-up is planned.

**Results:**

No significant effects (main or interaction) were observed for the outcomes. However, small positive differences were found for both outcomes for the participative learning intervention.

**Conclusions:**

These findings suggest that the 2 school-based interventions did not modify levels of physical activity or well-being within the period monitored. Change in physical activity may require more comprehensive individual behavioural intervention, and/or more system-based efforts to address wider environmental influences such as family, peers, physical environment, transport and educational policy.

**Trial registration number:**

ISRCTN82956355.

**Strengths and limitations of this study**

- We undertook what we believe to be the largest clustered randomised controlled trial of school based interventions designed to increase physical activity levels and feelings of well-being of students at the start of secondary school.
- Although the interventions did not seem to impact PA and well-being, they add to the growing body of evidence on ‘what works’ and what does not work for school-based interventions. Indeed in these times of economic austerity, and increasing focus on improving pupil attainment, data on unsuccessful change initiatives is vital to allow schools to focus on the optimal use of available resources to enhance students' health.
- There was an uneven return of data from the intervention arms although a state-of-the-art sensitivity analysis gives confidence in the conclusions.

## Background

It is now well understood that for many children and adolescents, regular participation in physical activity (PA) of at least moderate intensity is associated with improved physiological and psychological health.^1^ ^2^ Particular benefits, among others, include reduced cardiometabolic risk^3–6^ and lower odds of obesity.^7^ ^8^ In addition, longitudinal evidence suggests that PA behaviours established during childhood may carry over into adulthood.^9^ Recognising these health benefits and the need to promote PA during childhood, the current UK PA guidelines advise that all children and young people should engage in moderate-to-vigorous intensity PA (MVPA) for at least 60 min and up to several hours every day^10^ and include vigorous intensity activities, incorporating those that strengthen muscle and bone on at least 3 days a week. Further, the WHO additionally recommends that the majority of activity should be aerobic in nature, and accrued above and beyond non-recreational activities of daily living.^11^

Both self-reported and objectively measured data indicate that few secondary school-aged children engage in sufficient levels of MVPA. Hallal *et al*^12^ examined self-report data from 105 countries and estimated that 80.3% of 13–15-year olds do not engage in 60 min of MVPA per day or more. Recent accelerometer-derived data from the UK have led to estimates that both primary school children and older secondary school children engage in light intensity PA and sedentary time primarily, and only around 50% of boys and girls accrue 60 min or more of MVPA per day.^13^ ^14^ In addition to the low prevalence of children meeting PA recommendations, it is a pervasive finding in the literature that PA begins to decline during childhood, with this decline accelerating around adolescence.^15–17^ Change in PA during adolescence is associated with gender, perceived behavioural control, support for PA and self-efficacy.^18^ In addition, biological maturity has been shown to influence the PA decline in adolescents.^19^

Partly allied with insufficient levels of PA, there is concern in the research literature over the psychological well-being of school-aged children. Rates of mental ill health (eg, anxiety, depression and externalising problems) are relatively high in the UK. Approximately 1 in 10 of school-aged children at any one time have mental health problems.^16^ ^20^ ^21^ Furthermore, it is clear that there has been a considerable increase in the diagnosis and treatment of child and adolescent psychiatric disorders over the past 20 years.^21^ ^22^ While there is debate among researchers over the meaning of reported trends,^21^ in particular, whether this merely reflects changing diagnostic thresholds, and increased awareness and help seeking, rather than actual secular changes in mental health, there does appear to be evidence of increased difficulties for female adolescents. The picture is rather more uncertain with regard to males.^21–24^ Although for children the evidence base is not as well established as that for adults, it is important to note that a recent systematic review and meta-analysis^25^ and a review of reviews^6^ in school-aged populations have reported small-to-moderate effects for the benefits of PA on a number of psychological health outcomes including depression, anxiety, psychological distress, self-esteem/self-concept and emotional disturbance.^6^ ^25^ In addition, children who achieve recommended MVPA levels have been shown to report higher levels of well-being and health-related quality of life.^26^ Therefore, action to address the increasing prevalence of mental ill health and low levels of PA in school-aged children has been a public health priority for successive UK governments.^27^

A well-used and convenient setting in which to intervene is the school environment, due to the existing infrastructure, ability to maximise reach and possibility of curricular integration and longer term sustainability.^28^ A recent editorial published in the *BMJ* criticises school education policy in England for encouraging schools to maximise academic attainment while ignoring well-being and health.^29^ ^30^ The editorial suggests that schools should teach students the knowledge and skills required to promote their own mental and physical health, and recommends that such health-related education should be integrated into academic subjects as one means to reconcile competing educational demands.

Attempts to increase children's habitual PA behaviours have generally produced small effects.^31^ However, school-based initiatives have shown some potential^32^ ^33^ and a recent Cochrane review recommended the ongoing implementation of school-based PA interventions.^33^ In the light of such recommendations, the aim of the present study was to assess the impact of two education interventions on the levels of PA and self-reported well-being of year 7 (first year of secondary) school students in England.

The aims of the study were to assess:

1. The impact of two school-based education interventions, delivered separately or together, on mean minutes of accelerometer-measured MVPA per day.
2. The impact of the interventions, separately and together, on students’ levels of reported psychological well-being.

## Methods

### Study design

**Figure 1:**
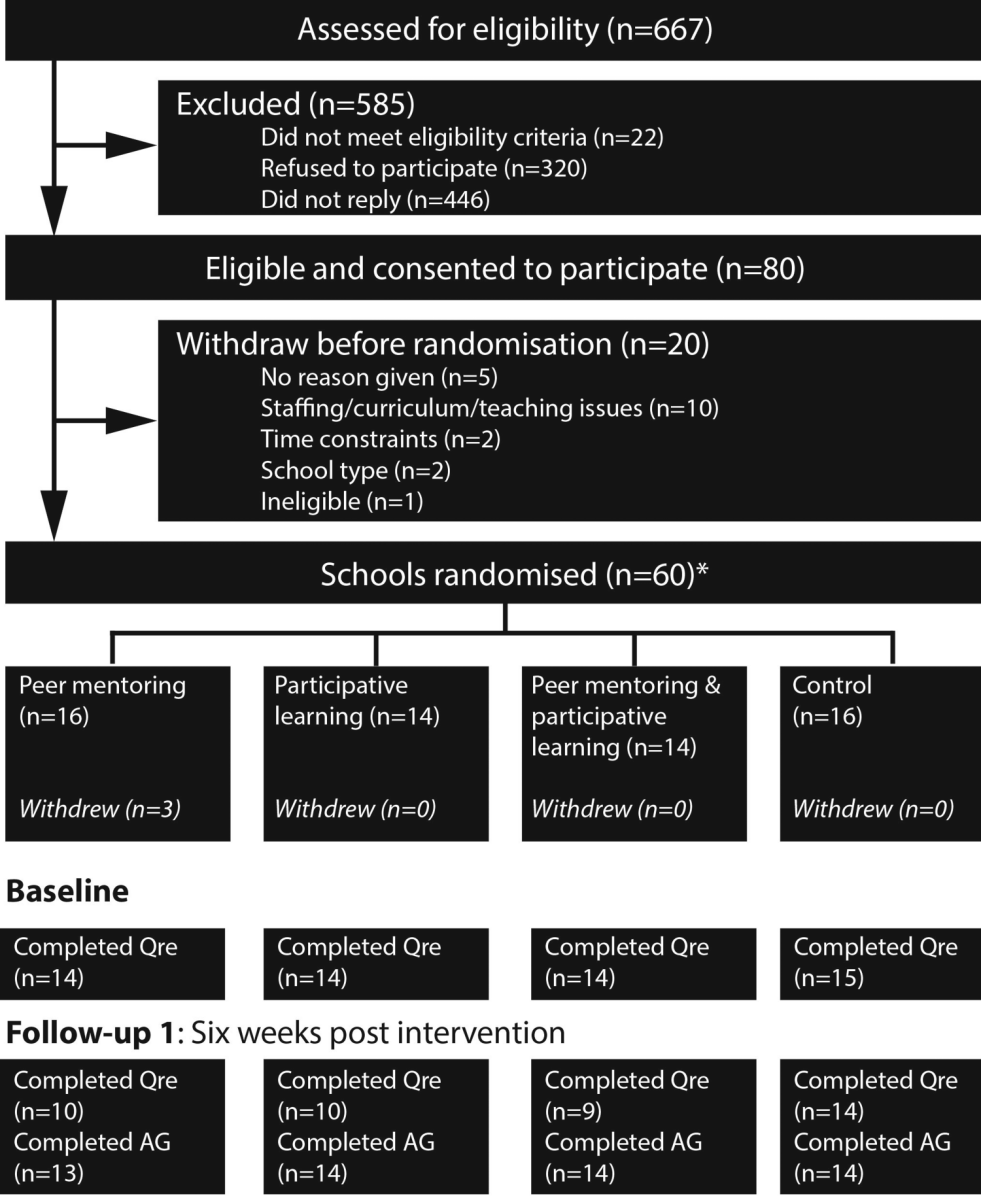
Flow of schools through study. Follow-up 1 represents end point data collected 6 weeks (on average) post-intervention. A ‘follow-up 2’ data collection has/is currently being taken and will be reported elsewhere. *Contrary to protocol, in which two control schools were allocated to intervention arms before baseline assessment. ‘Qre’ represents questionnaire data and ‘AG’ represents accelerometer data collection.

The research design (trial registration ISCRTN82956355) employed a 2×2 factorial stratified randomised controlled methodology with a ‘waiting list’ control group. In line with the power calculation, 60 secondary schools were recruited with one year 7 class and one year 9 class per school. The trial ran from October 2012 to July 2014. In the first year, year 7 students in different schools were allocated to one of three intervention arms or a waiting list control as shown in figure 1. Random allocation was at the school level, following recruitment. The students were unaware of their school's allocation until after the collection of baseline questionnaire data. Some middle schools, comprising only years 5 (age 9/10) to 8 (age 12/13), participated and therefore were paired with their corresponding high school to incorporate the year 9 (age 13/14) students needed for the peer-mentoring intervention. While we acknowledge that the different schools involved in the trial may have differing baseline and follow-up activity or mental health characteristics, due to the randomisation process, these differences are unlikely to have a significant effect. Further publications from the project will focus on examining how private and faith-based schools may influence students’ PA or psychological well-being.

The waiting list control group was scheduled to experience, in project year 2, the intervention that was shown to be most effective in the other schools in project year 1. All other participants were to be involved in the collection of follow-up data in project year 2.

### Eligibility and recruitment

All schools with years 7 and 9 students in the North of England were deemed to be eligible for inclusion. Schools were asked to confirm the availability of one year 7 and one year 9 class for the trial. The recruitment of schools was undertaken over an extended period (January 2012–December 2013) using letters to schools, talks to groups of head teachers, communications to local authority departments and by word of mouth. Initially, schools geographically closest to Durham University were contacted and the sampling frame was gradually extended to other adjacent areas until the required number of schools was reached. We invited 667 schools to participate and 80 agreed to take part. Twenty schools subsequently withdrew their consent before baseline for a variety of reasons (eg, Ofsted inspection, or staffing changes). As a result, the trial started with 60 schools.

Following receipt of each school's formal consent, parents/guardians of the children were sent a letter with information about the study and, as agreed by the Ethics Committee of Durham University School of Education (September 2012), an opt-out consent form for their child for the study evaluation measurements. Parents/guardians and children were given the opportunity to contact the research team to discuss the study and informed of their right to withdraw at any stage. The children were also given an information sheet and, at each of the measurement sessions, reminded of their right to withdraw. The flow of schools through the study is displayed in figure 1.

### Research setting

The majority of the schools involved (90%) were based in the North-East of England and the rest were from the North-West. Most were state schools and the sample also included: independent schools (8.3%) and special schools (3.3%). The National Curriculum was operating in all schools throughout the intervention and follow-up period, although independent schools and academies were not obliged to follow it. While there may be some variation in the application of the National Curriculum, the random allocation of schools to trial groups should mean that this will not have significantly affected the results.

### Randomisation

For each school, two z-score indicators, known to be related to PA, were calculated; one for the percentage of students who actively commuted to school (using data drawn from the School Census with supplementary data obtained from the local councils). The other was the Income Deprivation Affecting Children Index (IDACI; http://www.education.gov.uk/cgi-bin/inyourarea/idaci.pl). The schools were put into rank order on the basis of the sum of these z-scores. From this ranked list, the top four schools were grouped into one quartet, the next four into a subsequent quartet and so on. Each quartet was then randomly assigned to one of the four treatment groups. Any schools which did not fit into that list, because they were recruited at a later date, or because they were independent schools for which we could not obtain active travel data, were individually randomised to one of the four arms; this explains the uneven distribution of schools between the interventions. Randomisation was conducted by an independent statistician based at the Centre for Evaluation and Monitoring (CEM) at Durham University who was not involved in the trial.

### Interventions

Two interventions linked to the secondary school curriculum were implemented. The first, peer-mentoring, was linked to physical education (PE) classes in years 7 and 9 over a period of six weekly lessons, although in many cases, other periods in the school day were used. The second, ‘participative learning’ (PL), was designed to be run across six, weekly, year 7 geography lessons. Teachers were trained by the research team for the particular intervention in which they were participating and provided with intervention manuals and materials. Initial training typically took the form of a 2 h training session at Durham University, or in cases where this was not feasible, members of the research team delivered training in the schools concerned.

The PL intervention sessions were delivered in a classroom/computer room, and the peer-mentoring in a classroom, gym or any other suitable space available. Teachers were given flexibility over the delivery location, timing and frequency of intervention lessons. For example, for peer-mentoring, where there were no PE lessons during which the intervention could be delivered, teachers were given freedom to deliver the project during other time periods/lessons. Thus, while the intervention training and materials were standardised, their delivery was permitted to be flexible/adaptable to the school context in which they were being implemented.

### Peer-mentoring intervention

The peer-mentoring intervention employed a cross-age mentoring model which has been widely used in the learning of academic subjects, across a variety of disciplines with significant academic, attitudinal and motivational gains.^34^ ^35^ The older, year 9 students worked 1:1 with (where possible) same sex younger students (year 7), acting as peer mentors. The pairs met for weekly mentoring sessions of 20–30 min over a 6-week period, with a teacher providing overall class supervision. The year 9 students were provided with weekly training sessions by the intervention teachers in advance of each mentoring session. This sought to provide them with the requisite knowledge about PA, the behavioural techniques used and the skills/confidence to act as a mentor. This training included general principles of mentoring, as well as an overview of each weekly task.

In the weekly mentoring sessions, the student pairs worked through an intervention booklet which included learning tasks specific to the weekly focus, for example, identifying barriers to PA. The role of the year 9 mentor was to introduce the aim of each task, provide learning support (without giving answers) and to facilitate critical reflection by the mentee on his/her PA habits and how these could be improved. Following the first intervention week, the pairs set weekly activity goals for the year 7 student, who was encouraged to self-monitor their progress using an activity diary. During subsequent weeks, the pair reviewed goal progress, and discussed barriers/solutions to goal achievement where appropriate.

### PL intervention

This intervention used participatory application of Geographical Information Systems (GIS) technology in the classroom, supported by bespoke teaching materials created for the trial. The PL programme allowed children to collect and interpret data about their own day-to-day PA and, in the light of this, consider what factors in their environment affected their levels of activity. Research in the USA and the UK has shown that geographical setting is important for the level of PA young people take,^30^ ^36–38^ and the PL approach employed in the trial aims to use these ideas in the classroom to motivate change in activity levels. Community engagement in the production and/or use of geographic information aimed to empower participants to achieve personal or community goals.^39^ The PL intervention used here aimed to help students feel more informed and knowledgeable about where and how they could find opportunities for PA, which might result in enhanced self-efficacy and a greater sense of agency regarding the adoption of ‘healthy behaviours’.

A series of six lessons was devised for year 7 students. The fully resourced materials, linked closely to the National Curriculum, focused on neighbourhood influences on PA. Students participated by measuring their own level of PA and movement in space using accelerometers and Global Positioning System (GPS) units prior to the intervention. The lessons were designed to help students develop an understanding of how their environment may influence their health and well-being. They used mapping techniques in GIS software (https://www.arcgis.com/home) to identify where they had been most physically active, and to consider how and where they could be more active.

Details of the core intervention components can be found in online supplementary appendix tables 1–3. Further details concerning the precise nature of the peer-mentoring and PL approach can be found on the project website (http://www.move-project.org.uk).

### Controls

While the plan was to run the intervention against ‘business as usual’ controls, it is acknowledged that we have limited information on how their teaching programmes compared with the intervention schools.^40^ ^41^ Specific data were not collected from the control schools; however, they were generally following the National Curriculum and were not involved in activities which paralleled the peer-mentoring intervention or which employed GIS software in geography lessons.

### Modifications

Given the complexity and scale of the study, and the need for schools to continue with their routine functions, unfolding circumstances required us to modify certain aspects of our operations in ways that deviated from the original protocol. These are described below:

1. Recruitment of schools to the project proved difficult and the recruitment process was extended until sufficient schools were enlisted. As groups of four were recruited, they were allocated to treatments as described above (where possible). This continued so that the interventions were carried out over a full year.
2. Some schools had difficulties with operating the prescribed GIS software, ArcGIS Explorer Desktop (http://www.esri.com/software/arcgis/explorer-desktop) since their managed IT systems prohibited downloading of the software. It was decided mid-trial to use a web-based GIS package (ArcGIS Online—http://www.esri.com/software/arcgis/arcgisonline) for the schools involved in the PL approach. This switch prolonged the duration of the PL intervention by 6 weeks and as a result some schools did not complete the unit of work before the first outcome data were collected.
3. There were two occasions when the teachers were told the allocation before the initial questionnaire data had been collected; this was necessary in order that they could progress with timetabling the intervention. However, students were kept blind to the allocation.
4. Staffing constraints meant that accelerometer data were only collected at baseline for the PL group (as the data were required for the production of intervention content). Where possible, all schools had accelerometer data collected at follow-up.

### Fidelity to treatment

Fidelity was recorded in three ways. First (as reported in this paper), the researchers observed each intervention classroom at least once and subsequently rated the extent to which they felt that the lessons were adhering to the prescribed procedures. Second, after the interventions, teachers and students completed questionnaires which included specific and open-ended questions. Third, focus groups were run with samples of students.

### Data collection procedures

The sequence of data collection was as follows:

1. Administration of baseline questionnaires concentrating on participant demographics, measures of well-being and self-reported PA.
2. Interventions delivered and observations made.
3. Administration of postintervention questionnaires with measures of well-being and self-reported PA. Collection of accelerometer data as well as height and weight, not reported in this paper, for all year 7 students in the trial.
4. Administration of second postintervention questionnaires (‘follow-up 2’) approximately 12 months later. The data from this follow-up stage are not reported here.

### Outcome measures

#### Physical activity

PA was measured using three generations of ActiGraph accelerometers—GT1M, GT3X and GT3X+ (ActiGraph, Pensacola, Florida, USA). All instruments sampled PA at 30 Hz and the GT1M and GT3X units were programmed to store data at 10 s epochs. The GT3X+ stored raw acceleration data which were integrated to 10 s epochs postdownload for cross-generation comparability. Spatial location was recorded with a personal GPS receiver (Qstarz BT-Q1000X GPS, Qstarz International Co, Ltd, Taiwan) set to record at 10 s epochs. Participants were asked to wear the accelerometer on an elasticated belt over the right hip for seven consecutive days, with removal for swimming, bathing, sleeping and contact sports. Participants were provided with an instruction sheet. To promote compliance in wearing the instruments, a £10 gift voucher was offered to the one student in each class who returned the greatest volume of weekly wear time (preprocessed data).

Accelerometer data were downloaded using Actilife V.6.7.2 (ActiGraph, Pensacola, Florida, USA) and student-level daily data were generated and transferred to R (V.3.0.1) statistical software for further reduction. Non-wear time was defined as at least 60 min of consecutive zero counts with a 2 min interruption period. Extreme counts were defined as greater than 15 000 counts per minute. Activity counts were reduced into estimates of time in MVPA using the epoch-adjusted cut-points (383 counts per 10 s, as adjusted by Actilife) of Evenson *et al*.^29^

The number of hours of wear required to constitute a valid day was based on the 70/80 rule,^42^ that is (standard measurement day=(70th centile of sample wear time)×0.8). Resultant criteria were 406 min for weekdays and 351 min for weekends. Weekday estimates required at least 2 valid days; for weekend estimates at least 1 valid day; and for total week at least 3 days. To account for variation in wear time, moderate-to-vigorous intensity data were standardised to the sample weekday, weekend or total week average valid wear time (as appropriate) using the equation provided by Katapally and Muhajarine.^43^ The variable used in the analyses was the mean number of minutes of MVPA per day calculated from total week data.

#### Well-being

Self-report questionnaires KIDSCREEN-27^44^ were undertaken either online or on paper. The KIDSCREEN-27 is a 27-item questionnaire about perceived well-being in the domains of *physical well-being*, *psychological well-being*, *autonomy and parent relation*, *peers and social support*, and *school environment*.

#### Additional variables

The student-level data collected were matched with the National Pupil Database held by the Department for Education (http://www.adls.ac.uk/department-for-education/dcsf-npd/?detail). This provided data on ethnicity and socioeconomic position (IDACI score) and national key stage 2 standardised assessment test scores for the year 7 students when they were in year 6.

### Statistical analysis

In the analyses, the multilevel models nested students within schools; dummy variables identified the two interventions and an interaction term represented the two together, with the α level set at 0.05. The effects were allowed to vary across schools. Each outcome was analysed separately and the primary analysis compared PA and well-being outcomes in the year 7 students at first follow-up. For the accelerometer data, prior measures were not available for all arms but, because the data were collected throughout the year, adjustments were made for hours of daylight, by date of data collection.^45–47^ The well-being measures were adjusted for well-being as measured at baseline. For both outcomes, adjustments were made for sex, free school meal (FSM) eligibility and distance from home to school.

A small number of schools failed to produce some data (4 out of 56 for questionnaires, and 5 out of 56 for accelerometers) and, from the remaining schools, some student data were missing (30% and 40% for questionnaire and accelerometer data, respectively, out of 1325). For the MVPA outcome, an analysis using alternating logistic regression suggested that missingness of PA data at student level may be independent of school, but this did not hold for the KIDSCREEN questionnaire-based outcome.

## Results

### Population and data characteristics

Table 1 indicates that the study population was predominantly white British (>93%), with an almost even balance of males and females, and the proportion entitled to free school meals varied from 12% to 23% of participants in each of the four arms.

**Table 1:** Descriptive characteristics of study population

Characteristic	Peer-mentoring (n=322)	Participative learning (n=340)	Combined (n=337)	Control (n=392)
Age (year) mean (SD)	11.85 (0.46) (n=255)	11.84 (0.32) (n=320)	11.72 (0.31) (n=321)	11.79 (0.40) (n=339)
Sex (% boys)	52.50 (n=320)	50.88 (n=340)	48.96 (n=337)	46.68 (n=392)
Ethnicity	(n=269)	(n=307)	(n=288)	(n=314)
White British (%)	95.91	94.46	95.83	93.63
Other (%)	4.09	5.54	4.17	6.37
IDACI score mean (SD)	0.27 (0.16) (n=267)	0.23 (0.16) (n=287)	0.24 (0.20) (n=288)	0.20 (0.17) (n=313)
FSM (%)	23.05 (n=269)	17.59 (n=307)	17.36 (n=288)	12.10 (n=314)
School travel mode	(n=256)	(n=219)	(n=320)	(n=39)
Walk (%)	68.16	53.68	47.54	45.67
Cycle (%)	2.24	0.70	1.41	1.67
Car (%)	13.45	24.21	16.55	22.00
Bus/train (%)	16.14	21.40	34.51	30.67
KIDSCREEN data (%)	54.04	54.41	64.39	89.54
Received accelerometer (%)	65.84	95.88	82.20	87.76
Valid accelerometer data (% of those who received a device)	41.61	57.05	51.34	74.74
MVPA per day (min)	44.88 (SD=19.42)	52.68 (SD=22.70)	44.79 (SD=20.48)	45.11 (SD=19.38)
Valid accelerometer wear time (min)	737.35 (SD=112.50)	705.18 (SD=114.27)	692.61 (SD=112.85)	746.45 (SD=110.87)

IDACI, Income Deprivation Affecting Children Index; MVPA, moderate-to-vigorous intensity physical activity.

Proportions returning valid accelerometer data were: 42% in the peer-mentoring group, 57% in the PL group, 51% of the combined group and 75% of the control group.

Proportions that returned KIDSCREEN data were: 54% in the peer-mentoring group, 54% in the PL group, 64% of the combined group and 90% of the control group.

### Main intervention effects

The results depicted in table 2 indicate that, compared with the control group, the adjusted MVPA for the PL group was 3.23 (95% CI −2.87 to 9.35) minutes higher, the peer-mentoring group was 0.32 (−6.48 to 7.12) higher and the combination was 2.50 (−11.84 to 6.84) lower. None of these changes were statistically significant at the 5% level.

**Table 2:** Multi-level models for available cases and multiple imputation analysis of the moderate-to-vigorous intensity physical activity (MVPA) data

	Available cases		Multiple imputations	
Effect	unadjusted	Adjusted	Unadjusted	Adjusted
Fixed				
Intercept	45.88 (40.57 to 51.19)	21.45 (11.16 to 31.74)	46.88 (42.93 to 50.83)	20.52 (13.12 to 27.91)
Participative learning	7.92 (0.34 to 15.50)	3.23 (−2.87 to 9.35)	5.84 (−0.05 to 11.73)	2.64 (−1.66 to 6.94)
Peer mentoring	−0.02 (−8.23 to 8.20)	0.32 (−6.48 to 7.12)	0.75 (−5.26 to6.77)	0.20 (−4.20 to 4.61)
Both	−8.15 (−19.59 to 3.27)	−2.50 (−11.84 to 6.84)	−6.15 (−14.76 to2.45)	−2.10 (−8.52 to 4.32)
Daylight		1.48 (0.83 to 2.12)		1.55 (1.07 to 2.03)
Sex		11.77 (9.09 to 14.45)		12.54 (10.10 to 14.97)
FSM		4.43 (0.45 to 8.41)		4.22 (0.30 to 8.14)
Distance		−0.77 (−1.47 to −0.07)		−0.96 (−1.65 to −0.28)
Random				
School variance	80.30 (21.79)	39.20 (13.60)	42.71 (12.33)	42.70 (12.33)
Student variance	344.70 (17.88)	306.04 (17.03)	387.03 (20.76)	387.03 (20.76)
Intracluster correlation	0.19	0.11	0.10	0.10

The coefficients relate to the number of minutes of MVPA per day.

The figures in parentheses for the fixed part show the 95% CIs.

The figures in parentheses for the random part show the SEs.

For the adjusted KIDSCREEN global (total) scores in table 3, the scores for PL group were (−0.88 to 2.69) higher, the peer-mentoring group was a 0.70 (−2.57 to 1.17) lower and the combination was 1.93 (−4.98 to 0.70) lower. None of these changes were statistically significant at the 5% level.

**Table 3:** Multi-level models for available cases and multiple imputation analysis of the KIDSCREEN global score

	Available cases		Multiple imputation	
Effect	unadjusted	Adjusted	Unadjusted	Adjusted
Fixed				
Intercept	52.79 (51.56 to 54.02)	16.21 (11.85 to 20.57)	52.81 (51.72 to 53.90)	15.98 (12.24 to 19.71)
Participative learning	0.36 (−1.58 to 2.32)	0.90 (−0.88 to 2.69)	−0.66 (−2.30 to 0.98)	−0.26 (−1.54 to 1.02)
Peer mentoring	−1.06 (−3.041 to 0.92)	−0.70 (−2.57 to 1.17)	−1.19 (−2.88 to 0.49)	−0.55 (−1.89 to 0.78)
Both	−1.39 (−4.31 to 1.52)	−1.93 (−4.58 to 0.70	0.28 (−2.15 to 2.71)	−0.42 (−2.34 to 1.49)
Daylight		0.13 (−0.04 to 0.31)		0.15 (0.00 to 0.31)
Sex		−0.05 (−0.92 to 0.81)		−0.08 (−0.88 to 0.72)
FSM		−0.06 (−1.35 to 1.22)		0.24 (−0.92 to 1.403)
Distance		−0.01 (−0.24 to 0.23)		−0.04 (−0.25 to 0.17)
Pretest		0.67 (0.61 to 0.72)		0.66 (0.61 to 0.71)
Random				
School variance	3.12 (1.41)	2.31 (1.15)	2.25 (1.00)	1.30 (0.62)
Student variance	57.16 (2.73)	32.95 (1.84)	58.17 (2.70)	34.82 (1.73)
Intracluster correlation	0.05	0.07	0.04	0.04

The coefficients relate to the KIDSCREEN global score.

The figures in parentheses for the fixed part show the 95% CIs.

The figures in parentheses for the random part show the SEs.

### Sensitivity analyses

Sensitivity analyses, applied for both PA and well-being outcomes separately, used multiple imputation based on distance to school, gender, FSM and IDACI score, but did not substantively alter findings reported in the last two paragraphs (last columns in tables 1 and 2).

Further sensitivity analysis for ‘missing not at random’ (MNAR) was performed for KIDSCREEN scores alone using the MNAR model as proposed by Diggle and Kenward.^47^ The ‘Missing completely at random’ model assumed probability of dropout did not depend on baseline scores and post-test scores, while ‘missing at random’ assumed it depends only on the baseline score, but not the post-test score. The MNAR model assumed that the probability of dropout depends on both the baseline scores and the post-test scores.^46^ ^48^ The MNAR models indicated that students with low post-test score may have higher probability to dropout than those with high post-test score (see online supplementary appendix figure 1). However, the results from the weighted observed data showed no evidence to support positive impact of the interventions. This suggests that the missing data, were they observable, would be unlikely to change the main results and conclusions from this study.

Further multilevel models were run to assess whether there was any influence of the fidelity-to-treatment measures collected during visits to the school on either outcome measure. The ratings were on a 1–7 scale and designed to capture the extent to which the observed lessons corresponded to the planned intervention (1=not at all; 2;3;4=Around half; 5;6;7=Perfectly). For the PL intervention the mean was 5.28 (SD 1.62) and for the Peer-Mentoring the mean was also 5.28 (SD 1.16). No evidence was found of a link between the outcomes and the fidelity-to-treatment ratings.

For some schools, it was necessary to collect the post-test data before the PL intervention was complete and additional models looked for a link from the number of PL lessons carried out before the data were collected and the outcome measures. No such links were found.

## Discussion

### Main findings

This intervention study sought to modify the MVPA levels of children in their first year of secondary school, with the intention of enhancing feelings of well-being. We found no evidence of a significant effect of either intervention alone, or in combination, on these outcomes. When adjusting for covariates, the largest difference in PA was observed between the participative learning group and control, with these children doing an average of 3 min more of MVPA per day. Similarly the largest effect on total well-being scores was observed for the participative learning intervention.

### Comparison with previous findings

The findings of this paper add to the extant body of evidence^28^ suggesting that the impact of school-based intervention on MVPA is ‘small’. This leads to the conclusion that modest classroom initiatives do not have enough leverage to change children's levels of MVPA in substantive ways, at least in the period immediately after the intervention.

### Reasons for the finding

As noted above, the original plans had to be modified in some ways, and it is conceivable that this reduced power introduced artefacts into the data. It is also possible that the school sampling procedure introduced bias and/or that the interventions were not implemented well, although the fidelity data suggest that the interventions were carried out faithfully. Despite these provisos, if any effects had been substantial, we believe that these would have been identified in the data.

It is also possible that the interventions had a long-term impact which could not be detected with immediate post-test measures.

### Implications for practice

Modest school-based interventions designed to increase levels of PA are unlikely to have meaningful impact on MVPA or feelings of well-being.

As of September 2015, Ofsted, the national schools' inspection body, includes a judgement in their inspection framework on personal development, behaviour and welfare.^i^ This will examine the “extent to which schools and other providers are successfully supporting pupils to ‘gain knowledge of how to keep themselves healthy, including through exercising and healthy eating’.” Although this statement appears to miss the important link to action, it highlights the very pressing need for researchers in behavioural epidemiology to continue to work very closely with schools to trial PA and well-being change initiatives. It is hoped that these new inspection criteria may facilitate more proactive engagement between school leaders and research teams.

## Conclusions

Although Bonell *et al*^49^ call for greater attention to mental and physical health in schools, it is becoming increasingly clear that relatively modest classroom initiatives on the present scale are insufficient to change children's levels of PA. A multicomponent PA intervention targeting the home, school and wider neighbourhood environment may prove to be a more promising approach and might be usefully informed by the kinds of information collected through the interventions reported above. This could include structural changes to enable adolescents to travel safely in their environment and the provision of exclusion zones around schools where motorised transport is prohibited. Changes to educational policy to shift the accountability focus away from test results may also help. However, in identifying possible local solutions to low levels of PA among children, one must be cognisant of broader, and complex, ecosystemic influences^50^ that result in patterns of behaviour that are far from easy to address or resolve.

## Footnotes

i http://www.schoolfoodplan.com/wp-content/uploads/2015/02/APPG-School-Food-Letter.pdf.
